# Photo Quiz: A 69-year-old male presenting with a hepatic abscess

**DOI:** 10.1128/jcm.01430-25

**Published:** 2026-04-29

**Authors:** Andrew Walkty, Markus Stein, Heather Adam, James Karlowsky, John Embil

**Affiliations:** 1 Department of Internal Medicine, Section of Infectious Diseases, Max Rady College of Medicine, University of Manitoba 8664, Winnipeg, Manitoba, Canada; 2 Department of Medical Microbiology & Infectious Diseases, Max Rady College of Medicine, University of Manitoba 8664, Winnipeg, Manitoba, Canada; 3 Shared Health 366539, Winnipeg, Manitoba, Canada; Mayo Clinic Minnesota, Rochester, Minnesota, USA

**Keywords:** *Actinomyces israelii*, actinomycosis, hepatic abscess

## PHOTO QUIZ 

A 69-year-old male presented to a tertiary care hospital emergency department (Manitoba, Canada) with progressive generalized weakness and fatigue. His symptoms had been present for several months, and they became worse approximately 10 days prior to admission. The patient also complained of subjective chills and sweats over the preceding week. A review of systems was otherwise negative. His past medical history was significant for chronic lymphocytic leukemia, for which he was not currently receiving any treatment, hypertension, and gastroesophageal reflux disease. He had undergone a Whipple procedure 10 months prior to the current presentation for an intraductal papillary mucinous neoplasm. The only recent travel reported by the patient was to Alberta (Canada). He did not have any relevant exposures to animals. The patient denied any infectious contacts. He had no recent dental problems or dental work. He had done a bit of gardening earlier in the year.

**Fig 1:**
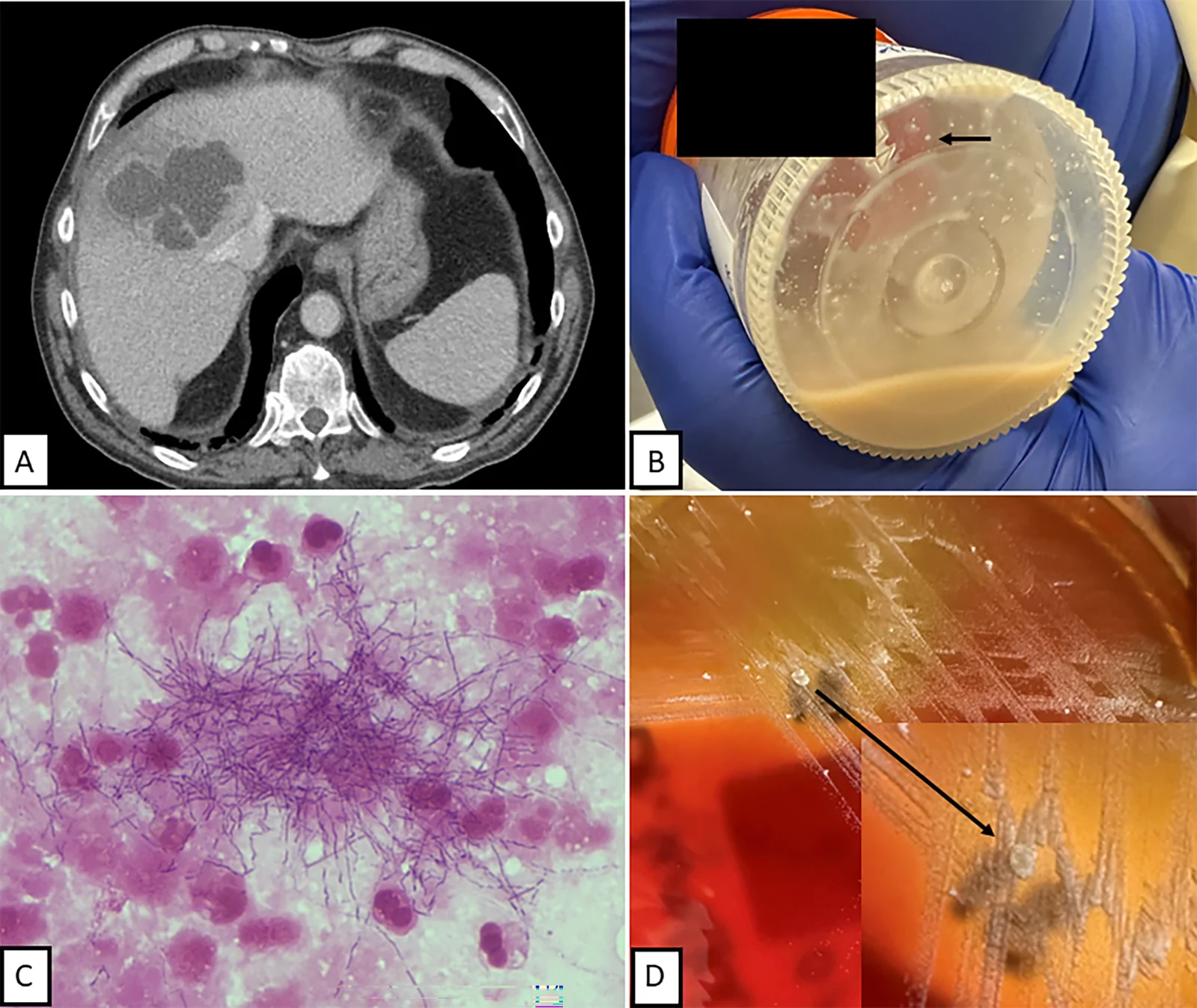
(**A**) A computed tomography scan of the patient’s abdomen, demonstrating a large intrahepatic abscess. (**B**) Purulent fluid obtained by aspiration of the abscess. (**C**) A Gram stain performed on the abscess fluid. (**D**) Colonies of the pathogen growing on *Brucella* blood agar after 5 days of incubation in an anaerobic atmosphere, with a magnified image (inset) illustrating the characteristic colony morphology (the black color seen in this image is ink on the other side of the plate).

On physical examination, the patient was afebrile but hypotensive, with a blood pressure of 63/45 mmHg. The remainder of the exam was unremarkable. Laboratory investigations revealed a peripheral white blood cell count of 20.5 × 10^9^/L (normal 4.5–11.0 × 10^9^/L) and a C-reactive protein of 117 mg/L (normal <5 mg/L). The emergency physician was concerned about the possibility of sepsis, given the degree of hypotension, and empiric therapy with piperacillin-tazobactam was initiated. A computed tomography scan of the abdomen to look for a source of infection demonstrated a large intrahepatic fluid collection, consistent with an abscess (Fig. 1A). A sample of the fluid (Fig. 1B) obtained under ultrasound guidance was submitted to the microbiology laboratory for aerobic and anaerobic bacterial culture, mycobacterial culture, and fungal culture.

A Gram stain of the fluid is presented in Fig. 1C. A pathogen was recovered on anaerobic bacterial culture. The characteristic colony morphology (seen after 5 days of incubation) is illustrated in Fig. 1D. What is your diagnosis?

## ANSWER TO PHOTO QUIZ

*Actinomyces israelii* was isolated on anaerobic culture of the abscess fluid, confirming the diagnosis of hepatic actinomycosis. No other organisms were recovered. Sulfur granules were seen on visual inspection of the abscess fluid (Fig. 1B, arrow). A Gram stain of the fluid demonstrated beaded and branching gram-positive rods, consistent with *Actinomyces* spp. (Fig. 1C). The characteristic molar tooth colony morphology of *A. israelii* was evident after 5 days of incubation on *Brucella* blood agar in an anaerobic atmosphere (Fig. 1D). Matrix-assisted laser desorption ionization-time of flight (MALDI-TOF) mass spectrometry (Bruker MALDI Biotyper System using Compass 4.1 (Build (100)) software and the MBT 12438 MSP library [last updated November 2023], Bruker Daltonics Ltd., East Milton, ON) failed to identify the organism. The organism was ultimately identified by 16S rRNA gene sequencing (99.7% similar to *A. israelii* type strain JCM 12964).

*Actinomyces* species are non-spore-forming gram-positive rods that grow best under anaerobic or microaerophilic conditions (1, 2). *A. israelii* can form branching filaments, and it may demonstrate a beaded appearance on Gram stain due to irregular staining (2). *Actinomyces* spp. are part of the normal human oral, gastrointestinal, and genitourinary flora (3, 4). As a consequence of phylogenetic analysis, several species previously belonging to the genus *Actinomyces* have been reassigned to other genera including *Schaalia*, *Gleimia*, and *Winkia* (3). Actinomycosis is an indolent, slowly progressive infection caused by bacteria belonging to the genus *Actinomyces* and related genera (3). It typically occurs secondary to a breach in the normal mucosal barriers (1, 3). Actinomycosis is characterized by progression across tissue boundaries, development of sinus tracts, chronicity, and relapse following a short course of therapy (3). The most common clinical presentations are orocervicofacial disease, thoracic disease, abdominal disease, and pelvic disease (3, 4). *A. israelii* is the species most frequently implicated as a cause of human actinomycosis (3). However, it should be recognized that the majority of actinomycosis cases are polymicrobial, with other recovered bacteria potentially serving as co-pathogens (3). Hepatic actinomycosis is uncommon (5). Among patients with hepatic actinomycosis, the lesion is most often solitary and may have a cystic/abscess-like appearance or a solid/tumor-like appearance on radiologic imaging (5).

A definitive diagnosis of actinomycosis can be made by recovery of *Actinomyces* spp. on culture of a specimen obtained from a normally sterile site, or via compatible histopathology (3). The demonstration of sulfur granules can be helpful. Sulfur granules represent a microscopic or macroscopic *in vivo* matrix of bacteria, calcium-phosphate, and other host materials (3). Macroscopic granules in a specimen will adhere to a container when pus is poured down the side, making them easier to visualize. They are typically yellow in color (hence the name sulfur granule) but can also be white, gray, or brown (3).

*Actinomyces* spp. are generally susceptible *in vitro* to beta-lactam antimicrobials including penicillin, ampicillin, ceftriaxone, piperacillin-tazobactam, and carbapenems, and resistant to metronidazole (6, 7). Penicillin is typically regarded as the drug of choice for actinomycosis, but ceftriaxone is considered a reasonable alternative (3, 8). Intravenous antimicrobial therapy is generally recommended for at least two to 6 weeks for patients with moderate-to-severe disease. If clinical improvement occurs, the patient may then be stepped down to oral penicillin or amoxicillin for at least 6–12 months, with the total duration of therapy guided by clinical and radiographic response (3, 8). When co-pathogens are isolated on culture, it is generally suggested that these also be covered with an appropriate antimicrobial during the initial treatment course (3, 8). The patient described here was discharged on once daily intravenous ceftriaxone for convenience. When seen in follow-up several weeks later, he had improved clinically and radiographically.
